# Impact of Freezing on the Nanoarchitecture and Techno‐Functional Properties of Camel Myofibrillar Proteins: Insights From Atomic Force Microscopy

**DOI:** 10.1002/fsn3.71668

**Published:** 2026-04-27

**Authors:** Ahmed‐Laloui Hamza, Rahmani Abderrahmen, Cherb Nora, Bendjaballah Sandra, Ramdani Nacira, Sajid Maqsood, Seid Mahdi Jafari

**Affiliations:** ^1^ Animal Production Team, Biotechnology and Agriculture Division; Biotechnology Research Center Constantine Algeria; ^2^ Environment Biotechnology Division; Biotechnology Research Center Constantine Algeria; ^3^ Industrially Biotechnology Division; Biotechnology Research Center Constantine Algeria; ^4^ Regional Veterinary Laboratory of El Oued National Institute of Veterinary Medicine El Oued Algeria; ^5^ Department of Food Science, College of Agriculture and Veterinary Medicine United Arab Emirates University Al‐Ain UAE; ^6^ Department of Food Materials and Process Design Engineering Gorgan University of Agricultural Sciences and Natural Resources Gorgan Iran; ^7^ Halal Research Center of IRI, Iran Food and Drug Administration, Ministry of Health and Medical Education Tehran Iran

**Keywords:** camel meat, frozen storage, protein nanostructure, SDS‐PAGE, thermal treatment

## Abstract

This study provides the first comprehensive nanoscale and functional evaluation of camel myofibrillar proteins subjected to long‐term frozen storage. A combination of atomic force microscopy (AFM), biochemical assays, and electrophoresis was employed to investigate structural integrity, gelation capacity, and protein stability in *Biceps femoris* and *Longissimus lumborum* muscles, analyzed in both fresh and preserved states (−20°C for 1 year), including post‐heating conditions simulating gel formation. AFM revealed significant topographical alterations in preserved samples, particularly in *L. lumborum*, including increased surface roughness, disrupted fibrillar architecture, and enlarged cross‐sectional dimensions. These nanoscale changes were accompanied by increased solubility and surface hydrophobicity, alongside significantly reduced sulfhydryl group availability, which results in oxidation, aggregation, and compromised gel matrix stability. In contrast, fresh samples retained a compact, homogeneous network with smoother surfaces, reflecting superior molecular organization and functional integrity. SDS‐PAGE confirmed progressive degradation of key myofibrillar proteins—MHC, actin, α‐actinin, troponin T, and MLC, with preserved *L. lumborum* exhibiting additional low‐molecular‐weight bands, indicative of extensive proteolysis. These structural disruptions correlated with reduced gel cohesiveness and potentially diminished water‐holding capacity, key quality attributes in muscle‐based food systems. Overall, this study demonstrates that prolonged freezing induces pronounced nano‐structural and functional destabilization of myofibrillar proteins, with responses that differ according to muscle‐specific composition.

## Graphical abstract

Schematic overview of the extraction of camel myofibrillar proteins from *Longissimus lumborum* (*LL*) and *Biceps femoris* (*BF*) muscles and the evaluation of freezing‐induced changes. The study integrates techno‐functional analyses (protein solubility, surface hydrophobicity, reactive sulfhydryl groups, SDS–PAGE) with nanoarchitectural characterization using atomic force microscopy to assess the impact of frozen storage on camel muscle proteins.

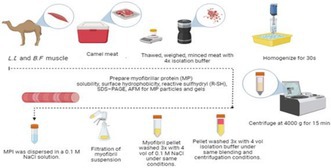

## 1 Introduction

Meat serves as a primary source of high‐quality dietary proteins globally. Among various animal species, camel meat represents a valuable yet underutilized protein source, particularly in arid and semi‐arid regions where camels are prevalent (Abduku et al. 2025). In addition to its nutritional benefits, consumer acceptance of camel meat products is largely determined by sensory attributes e.g., color, texture, and flavor, which are closely linked to the structure and functionality of muscle proteins (Choi and Kim 2009; Wu et al. 2024). Myofibrillar proteins (MPs), primarily composed of myosin, actin, tropomyosin, and troponins, constitute approximately 60%–70% of total muscle proteins (Xiong 1994). These proteins not only drive muscle contraction but also play a pivotal role in determining the functional properties of processed meat products, particularly gelation, water‐holding capacity (WHC), and textural characteristics (Sun and Holley 2011). Myosin, a key component, forms the structural backbone of protein gels, while actin supports gel integrity and contributes to tenderness through its interactions with myosin (López‐Bote 2017; Wang et al. 2020). Given their functional significance, MPs are central to the functional performance of muscle‐based foods, especially in terms of their texture, WHC, and ability to form stable gel matrices. These macroscopic traits are governed by the nanoscale organization of protein assemblies, which are highly sensitive to environmental stressors e.g., freezing and thermal processing (Foegeding and Davis 2011).

Freezing and long‐term frozen storage are widely used preservation strategies aimed at extending shelf life and maintaining the microbiological and sensory quality of meat (Coombs et al. 2017; Coria‐Hernández and Meléndez‐Pérez 2024; Li et al. 2018). However, these processes can also promote protein denaturation, oxidation, and aggregation, ultimately leading to diminished structural integrity and impaired functional performance (Arokiyaraj et al. 2024; Xu et al. 2020). Understanding these molecular changes, particularly at the nano/microscale levels, is essential for optimizing preservation protocols and improving the quality of processed meats. To investigate such nanoscale modifications, a range of analytical techniques, including X‐ray crystallography, Raman spectroscopy, circular dichroism, and electron microscopy, have been employed to elucidate protein conformation and molecular interactions (Guo et al. 2017; Jiang et al. 2025; Ringe and Petsko 1986; Wang, Sun, et al. 2017; Xu et al. 2011). Among these, Atomic Force Microscopy (AFM) has recently emerged as a particularly powerful high‐resolution imaging tool. AFM enables three‐dimensional (3D) visualization of protein surface topographies at the nanoscale and provides both qualitative and quantitative information on protein conformation, aggregation, and surface morphology, all without the need for extensive sample preparation (Arredondo‐Tamayo et al. 2023; Ding et al. 2019; Emam‐Djomeh et al. 2020; Obeid and Guyomarc'h 2020; Shi et al. 2019a). In food proteins, AFM has been successfully applied to characterize proteins from milk, fish, and various meats (Foegeding and Davis 2011; Shi et al. 2019b). Notably, it has proven effective in profiling volumetric and morphological transitions that directly influence protein functionality and food quality (Fuentes‐Perez et al. 2013).

However, despite its potential, AFM remains underutilized in camel meat research, particularly in the structural characterization of MPs, thereby representing a critical knowledge gap in the current literature. This study aims to fill this gap by employing AFM to investigate the nanoscale architecture and thermal behavior of MPs extracted from *Biceps femoris* (*BF*) and *Longissimus lumborum* (*LL*) muscles of camel, comparing fresh and preserved samples (frozen at −20°C for 1 year). Complementary biochemical assays, solubility, surface hydrophobicity (Sur_H_), and reactive sulfhydryl (R‐SH) content, alongside electrophoresis profiling, were also conducted to correlate nanoscale structure with functional and molecular stability. This integrated analytical framework provides novel insights into the structural transformations induced by freezing, contributing to the development of better preservation and processing strategies for camel meat and other underutilized protein resources.

## 2 Materials and Methods

### 2.1 Ethics Statement

Camel muscle samples used in this study were obtained post‐mortem from adult dromedary camels slaughtered for routine commercial meat production at the licensed Municipal Slaughterhouse of El Oued (Algeria). No animal was slaughtered for research purposes. Therefore, ethical committee approval was not required under Algerian legislation (Law 88‐08; Executive Decree 95‐363; Interministerial Order of 21/11/1999). All slaughtering procedures adhered to Algerian halal regulations. All procedures adhered to good animal handling and welfare practices in line with international standards (Directive 2010/63/EU).

### 2.2 Materials

Samples were collected from *Longissimus lumborum* (*LL*, lumbar region) and *Biceps femoris* (*BF*, posterior compartment of the thigh) muscles (“Recommended terminology for the muscle commonly designated ‘longissimus dorsi’,” Recommended Terminology for the Muscle Commonly Designated ‘longissimus dorsi’ 1990) from seven young male Sahraoui dromedaries ( *Camelus dromedarius* , 2–3 years old, live weight 125–250 kg) (*n* = 7 independent carcasses). In accordance with Algerian regulations, only young males were used, as the slaughter of females is prohibited. These young camels, popularly known in the region as *Hashi*, were clinically healthy and slaughtered in a commercial abattoir in Oued Souf, Algeria, following halal procedures after a 12 h feed withdrawal (Al‐Owaimer et al. 2014; Smili et al. 2022). Carcasses were kept at 12°C until 12 h postmortem, and subsequently chilled at 4°C for 24 h to ensure proper conditioning before sampling.

After chilling, *LL* muscles were excised from the lumbar vertebrae and *BF* muscles from the hind limb of each carcass. For each muscle type, tissues from the seven carcasses were diced into approximately 15 g pieces and thoroughly mixed separately (*LL* pooled independently; *BF* pooled independently) to generate two distinct composite muscle batches representative of the sampled population.

Following homogenization within each muscle type, the pooled material was subdivided into two preservation treatments: fresh and long‐term frozen. Each treatment consisted of 12 equal portions (~150 g each), vacuum‐sealed in Cryovac bags.

Fresh samples were refrigerated at 4°C for 24 h and analyzed immediately thereafter, while preserved samples were frozen at −20°C for 1 year and thawed at 4°C for 16 h prior to analysis to evaluate long‐term stability (Park et al. 2007; Setyabrata and Kim 2019). For each muscle‐treatment combination, 3–5 independent experimental trials were conducted. In each trial, one randomly selected vacuum‐sealed portion derived from the corresponding pooled muscle batch was used for analysis. Thus, subsequent analytical measurements represent technical/experimental replications performed on composite muscle material.

### 2.3 Preparation of Myofibrillar Proteins

Myofibril isolations were conducted following the procedures described by Park et al. (2007) and Hu et al. (2021). All preparation steps were carried out below 4°C. Thawed and minced muscle tissue from *LL* and *BF* muscles were mixed with 4 volumes (w/v) of an isolation buffer consisting of 3.9 mM KH_2_PO_4_, 6.1 mM KHPO_4_, 0.1 M NaCl, 2 mM MgCl_2_, and 4 mM EDTA at pH 7.0. The mixture was homogenized for 30 s in the T10 Basic S25 homogenizer (IKA Co., Germany). Next, the muscle homogenate was centrifuged at 4000 *g* for 15 min, and the supernatant was discarded. The resulting pellet was washed three times with 4 volumes of the same isolation buffer using identical blending and centrifugation conditions as described above. Subsequently, the myofibril pellet underwent three additional washes with 4 volumes of 0.1 M NaCl under the same conditions, except for the last wash, where MP suspension (MPS) was filtered through four layers of cheesecloth to remove connective tissue. MP isolate (MPI) was dispersed in a solution containing 0.1 M NaCl and stored at 4°C overnight for further analysis. Additionally, the samples stored at −20°C for 1 year followed the same procedures as described above for MP isolations.

### 2.4 Analysis of Protein Solubility

MPS was centrifuged at 8000 *g* for 15 min. Protein concentration was determined using the Bradford method with a spectrophotometer (Thermo Scientific Helios Zeta UV–Vis). Then, protein solubility (S%) was defined as the ratio of the protein concentration in the supernatant (*C*_*after*_) to that of the original MPS (*C*_*before*_) (Wang, Xiong, and Sato 2017); calculated by Equation (1):

(1) $$S\%=\frac{C_{\text{after}}\;}{C_{\text{before}}}\times 100$$

### 2.5 Determination of Surface Hydrophobicity

Surface hydrophobicity (Sur_H_) was determined using bromophenol blue (BPB) as a hydrophobic probe according to Chelh et al. (2006) and Zheng et al. (2019), with some modifications (Chelh et al. 2006; Zheng et al. 2019). MPS was adjusted to a concentration of 2 mg/mL. Then, 20 μL of BPB solution (1 mg/mL) was added to 1 mL MPS and stirred for 20 min at room temperature. The mixture was then centrifuged at 2000 *g* for 15 min. The absorbance of the supernatants was measured at 595 nm (*A*_*sample*_). For the determination of the control absorbance (*A*_*control*_), 0.1 M NaCl was used to replace MPS. Sur_H_ was expressed by the concentration of BPB bound, which was calculated using Equation (2):

(2) $$\mathrm{BPB}\;\text{bound}\;\left(\left(\mathrm{\mu g}\right)/\mathrm{mg}\right)=\frac{20x\left(A_{\text{CONTROL}}-A_{\text{SAMPLE}}\right)}{A_{\text{CONTROL}}x2}$$

### 2.6 Reactive‐Sulfhydryl Contents

R‐SH contents were determined according to Ellman et al. (1961). For this, 5 mL diluted MPS was mixed with 100 μL of 5, 5′‐dithiobis‐2‐nitrobenzoic acid (DTNB, 10 mM, pH 8.0). The mixtures were placed in the dark for 30 min at room temperature before centrifugation. The absorbance of the supernatants was measured at 412 nm. The concentration of diluted MPS was determined by Bradford method. R‐SH content was calculated using the molar extinction coefficient (*ϵ*) = 13,700 M^−1^ cm^−1^ based on Equation (3):

(3) $$C_{0=\frac{A}{\epsilon }\times D}$$

where: *C*_0_ = original concentration, *A* = absorbance at 412 nm, and *D* = dilution factor.

### 2.7 Sodium Dodecyl Sulfate–Polyacrylamide Gel Electrophoresis (SDS–PAGE)

SDS‐PAGE was performed to determine the structural changes of MPs according to (Maqsood et al. 2015, 2016, 2018) with some modification. Firstly, SDS‐PAGE running buffer (25 mM Tris, 250 mM glycine, 0.1% (w/v) SDS) and sample loading buffer (1 M Tris–HCl buffer, pH 6.8, 4% (w/v) SDS, 20% (v/v) glycerol, 0.2% (w/v) BPB, 10% β‐mercaptoethanol) were prepared. A protein standard (17–250 kDa) was used as the protein marker. The mixture was heated in boiling water (95°C) for 5 min to completely dissolve the protein and then chilled at room temperature. A concentration of 5 mg/mL of samples (10 μL; including loading buffer) was loaded onto each well. SDS‐PAGE with a 4% acrylamide stacking gel and a 12% acrylamide resolving gel was used. Coomassie Brilliant Blue R‐250, ethanol, and acetic acid were used in the staining and destaining processes. The resulting gels were imaged using the Bio‐Rad Gel Doc XR+ Imaging System (Bio‐Rad, Hercules, California). Image Lab version 5.1 software from Bio‐Rad was used to analyze gel images.

### 2.8 Atomic Force Microscopy

The surface morphology and nanoscale structural features of MPs and MP gels were assessed under ambient conditions using AFM (Bruker Multimode 8, Veeco Co., Plainview, USA), in accordance with (Hu et al. 2021; Mills et al. 2001), with minor modifications. Following best practices established in recent nanoimaging protocols (Ding et al. 2019; Shi et al. 2019a), AFM allowed visualization of protein surface structure with molecular‐scale resolution. For protein particles, 50 μL of MP solution (1 mg/mL) was deposited onto freshly cleaved mica and allowed to dry for 15 min at room temperature. The samples were then rinsed with ultrapure distilled water to remove unbound materials and air‐dried. For MP gels formation, MPS was thermally treated in a water bath at 70°C for 1 h, followed by natural drying at room temperature to prepare gel‐phase samples suitable for AFM scanning. Imaging was conducted using the Nanoscope 9.0 software in tapping mode, maintaining a low contact force (< 1 nN) to minimize sample deformation. Scanning was performed at resolutions of 2.0 × 0.2 μm and 650 × 650 nm, with a scan frequency of 0.996 Hz. Data acquisition was complemented by quantitative image analysis using Nanoscope Analysis 1.5 (Bruker Co., Santa Barbara, CA), enabling height profile assessments and morphological quantification such as surface roughness, fibril width, and aggregate diameter.

### 2.9 Statistical Analysis

All statistical analyses were performed using XLSTAT software (Version 10.0; Addinsoft, New York, USA).

The experimental design was based on a pooled‐sample strategy. For each muscle type (*Longissimus lumborum* and *Biceps femoris*), tissues obtained from seven independent camel carcasses (*n* = 7 biological sources) were diced, thoroughly mixed, and homogenized to generate a composite muscle batch representative of the studied population. *Longissimus lumborum* (*LL*) and *Biceps femoris* (*BF*) were pooled separately and remained distinct composite experimental units. Following homogenization, each pooled muscle batch was subdivided into two preservation treatments: fresh (24 h at 4°C) and long‐term frozen (−20°C for 1 year). Because the freezing treatment was applied to portions derived from the same pooled muscle material, the experimental unit corresponded to the pooled muscle batch rather than individual animals.

Accordingly, the design was not structured as a fully factorial model with independent biological replication per factor combination. Therefore, interaction terms (muscle × preservation) were not formally tested to avoid pseudoreplication and violation of independence assumptions.

Statistical comparisons were structured according to the experimental hierarchy:

- Paired *t*‐tests were used to evaluate the effect of freezing within each muscle type (fresh vs. frozen *LL*; fresh vs. frozen *BF*).
- Independent *t*‐tests were applied to compare *LL* and *BF* within the same preservation state (fresh *LL* vs. fresh *BF*; frozen *LL* vs. frozen *BF*).

For each muscle‐treatment combination, 3–5 independent experimental trials were performed using randomly selected vacuum‐sealed portions derived from the pooled muscle material. All data are expressed as mean ± standard error of the mean (SEM), and statistical significance was set at *p* < 0.05.

## 3 Results

### 3.1 Changes in Myofibrillar Protein Properties

To evaluate the impact of long‐term frozen storage on myofibrillar protein properties, solubility, surface hydrophobicity, and reactive sulfhydryl contents were measured in both muscle types, and the results are summarized in Table 1. In the fresh state, protein solubility did not differ significantly between *BF* (2.84 ± 0.32) and *LL* (2.90 ± 0.92), as indicated by identical lowercase letters within the fresh group (*p* > 0.05). However, freezing for 1 year at −20°C resulted in a marked and significant increase in solubility in both muscles (*p* < 0.05), reaching 12.20 ± 1.01 in *BF* and 11.69 ± 1.66 in *LL*, as reflected by different uppercase letters within each muscle type (fresh vs. preserved). No significant difference was observed between preserved *BF* and *LL* samples (identical lowercase letters within the preserved group, *p* > 0.05).

**TABLE 1:** The solubility, surface hydrophobicity, and reactive‐sulfhydryl group of MP particles.

Parameter	Fresh *BF*	Preserved *BF*	Fresh *LL*	Preserved *LL*
Protein solubility	2.84 ± 0.32^Ba^	12.20 ± 1.01^Aa^	2.90 ± 0.92^Ba^	11.69 ± 1.66^Aa^
Surface hydrophobicity	4.89 ± 0.37^Ba^	6.78 ± 0.44^Ab^	3.17 ± 0.19^Bb^	7.19 ± 0.013^Aa^
Reactive‐Sulfhydryl contents (R–SH)	65.54 ± 4.22^Aa^	41.72 ± 2.85^Ba^	68.49 ± 2.42^Aa^	36.58 ± 3.38^Bb^

*Note:* Lowercase letters indicate significant differences between the two types of muscles within the same preservation group (*p* < 0.05). Uppercase letters denote significant differences between the same types of muscles within each preservation group (*p* < 0.05).

Surface hydrophobicity (SurH) showed differences related to both muscle and conservation. In fresh samples, *BF* showed significantly higher SurH (4.89 ± 0.37) than *LL* (3.17 ± 0.19), as indicated by different lowercase letters within the fresh group (*p* < 0.05). After frozen storage, SurH increased significantly in both muscles (different uppercase letters within each muscle type, *p* < 0.05), get to 6.78 ± 0.44 in *BF* and 7.19 ± 0.013 in *LL*. In preserved samples, *LL* displayed significantly higher SurH than *BF* (different lowercase letters within the preserved group, *p* < 0.05).

Reactive sulfhydryl (R–SH) contents did not differ significantly between fresh *BF* (65.54 ± 4.22) and fresh *LL* (68.49 ± 2.42), as indicated by identical lowercase letters (*p* > 0.05). In contrast, frozen storage significantly reduced R–SH levels in both muscles (different uppercase letters within each muscle type, *p* < 0.05), decreasing to 41.72 ± 2.85 in *BF* and 36.58 ± 3.38 in *LL*. In preserved samples, *BF* retained significantly higher R–SH content than *LL* (different lowercase letters within the preserved group, *p* < 0.05).

### 3.2 SDS‐PAGE of Myofibrillar Proteins

**FIGURE 1:**
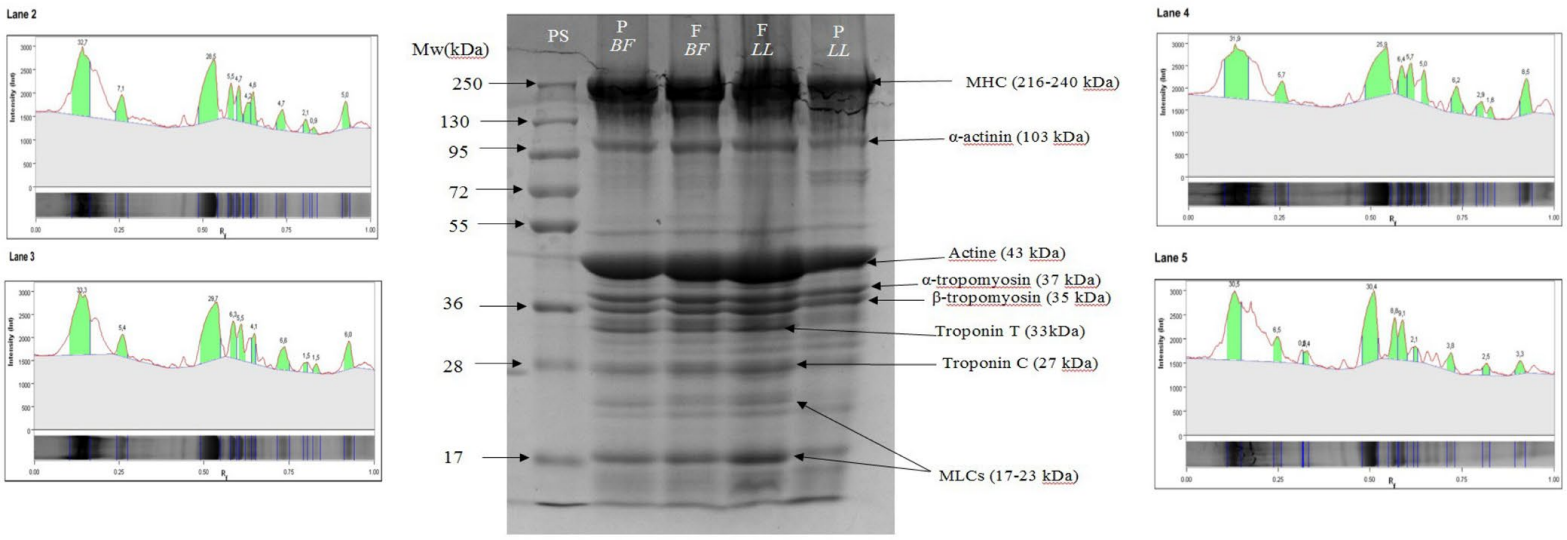
SDS–PAGE profile of myofibrillar proteins extracted from camel *Biceps femoris* (BF) and *Longissimus lumborum* (LL) muscles under fresh (F) and preserved (P, 1 year at −20°C) conditions. PS, protein standard. Major bands were tentatively assigned, based on molecular weight markers and previous reports in camel muscle (Maqsood et al. 2015, 2016, 2018).

Electrophoretic profiles of myofibrillar proteins extracted from *Biceps femoris* (*BF*) and *Longissimus lumborum* (*LL*) muscles of *Camelus dromedarius* under fresh and preserved (−20°C for 1 year) conditions are presented in Figure 1. Major bands corresponding to myosin heavy chain (MHC, 216–240 kDa), α‐actinin (103 kDa), actin (43 kDa), troponin T (TT, 33 kDa), and myosin light chains (MLC, 17–23 kDa) were identified according to molecular weight markers and previous reports (Khatri and Huff‐Lonergan 2023; Maqsood et al. 2015, 2016; Wu et al. 2024).

In *BF* muscle, long‐term frozen storage induced limited modifications. Slight reductions were observed for MHC (3.78%) and actin (5%), whereas MLC (17 kDa) and MLC (23 kDa) decreased by 18% and 73%, respectively. α‐Actinin and TT remained relatively stable. In contrast, preserved *LL* samples showed substantial degradation of several major proteins. Reductions reached 35% for MHC, 24% for α‐actinin, and 21% for actin, while TT and MLC (17 kDa) decreased by 72% and 73%, respectively. In addition, new bands between 75 and 85 kDa appeared exclusively in preserved *LL* samples, suggesting fragmentation of high‐molecular‐weight proteins. The magnitude and distribution of these changes indicate a more extensive modification of myofibrillar proteins in *LL* than in *BF* under identical storage conditions.

### 3.3 Topology Changes of Myofibrillar Proteins and Myofibrillar Protein Gels

#### 3.3.1 Nanoscale Structural Characterization of Myofibrillar Proteins

**FIGURE 2:**
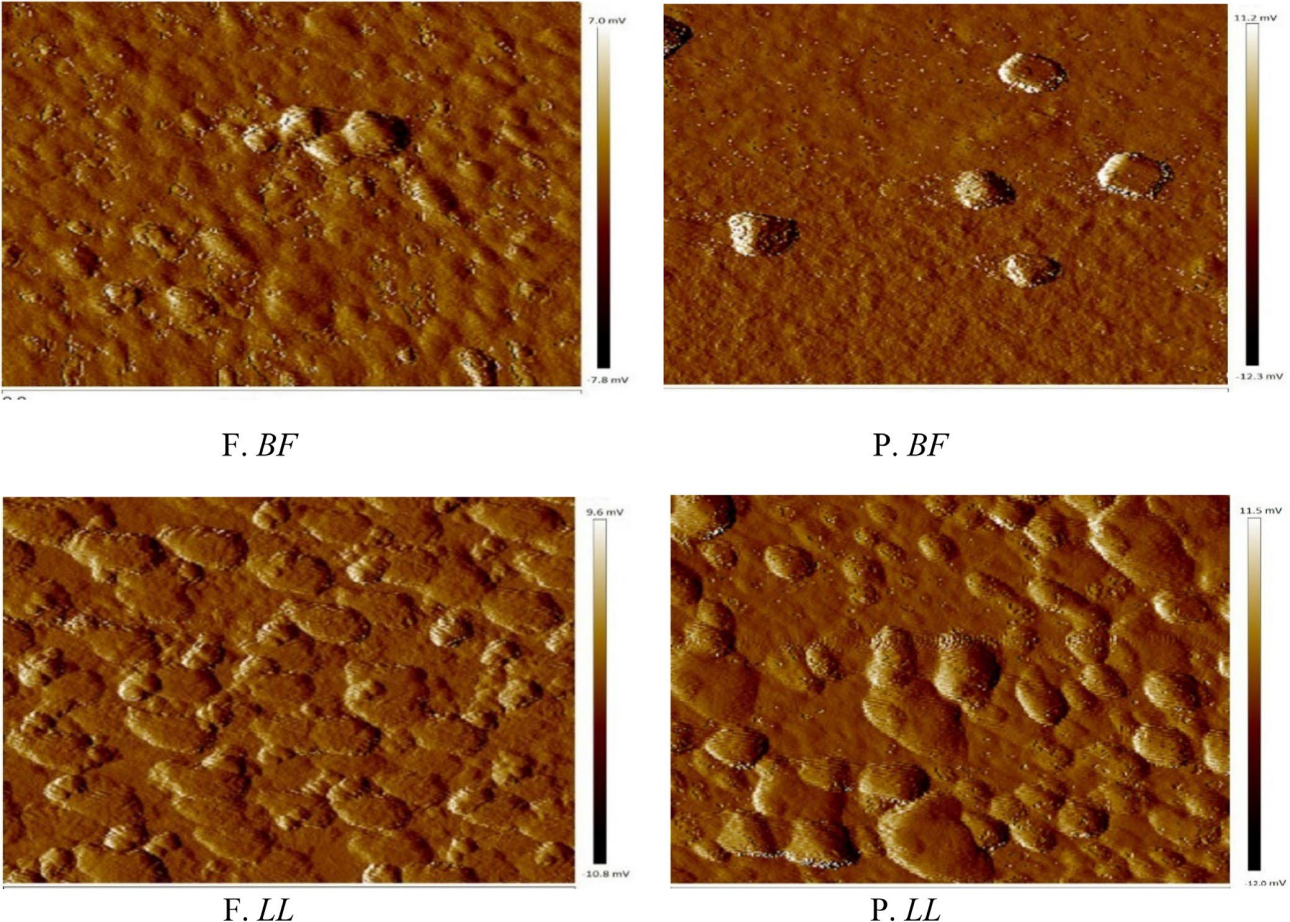
Atomic force microscopy (AFM) images of myofibrillar protein particles from *Biceps femoris* (BF) and *Longissimus lumborum* (LL) muscles in fresh (F) and preserved (P) states (stored at −20°C for 1 year).

AFM topographical images of myofibrillar proteins obtained under fresh (F) and preserved (P) conditions are shown in Figure 2. In fresh *BF* (F. *BF*) and fresh *LL* (F. *LL*), protein particles appeared as compact, elevated convex domains with smooth and continuous surfaces. These structures exhibited relatively homogeneous distribution and well‐defined boundaries, indicating preserved structural organization (Figure 2, F. *BF* and F. *LL*). After long‐term frozen storage (−20°C for 1 year), clear morphological alterations were observed in both preserved BF (P. *BF*) and preserved LL (P. *LL*). The surfaces exhibited irregular aggregation, concave depressions, disrupted alignment, and increased roughness compared to their fresh counterparts (Figure 2, P. *BF* and P. *LL*). These alterations were visually more pronounced in *LL* than in *BF*.

**FIGURE 3:**
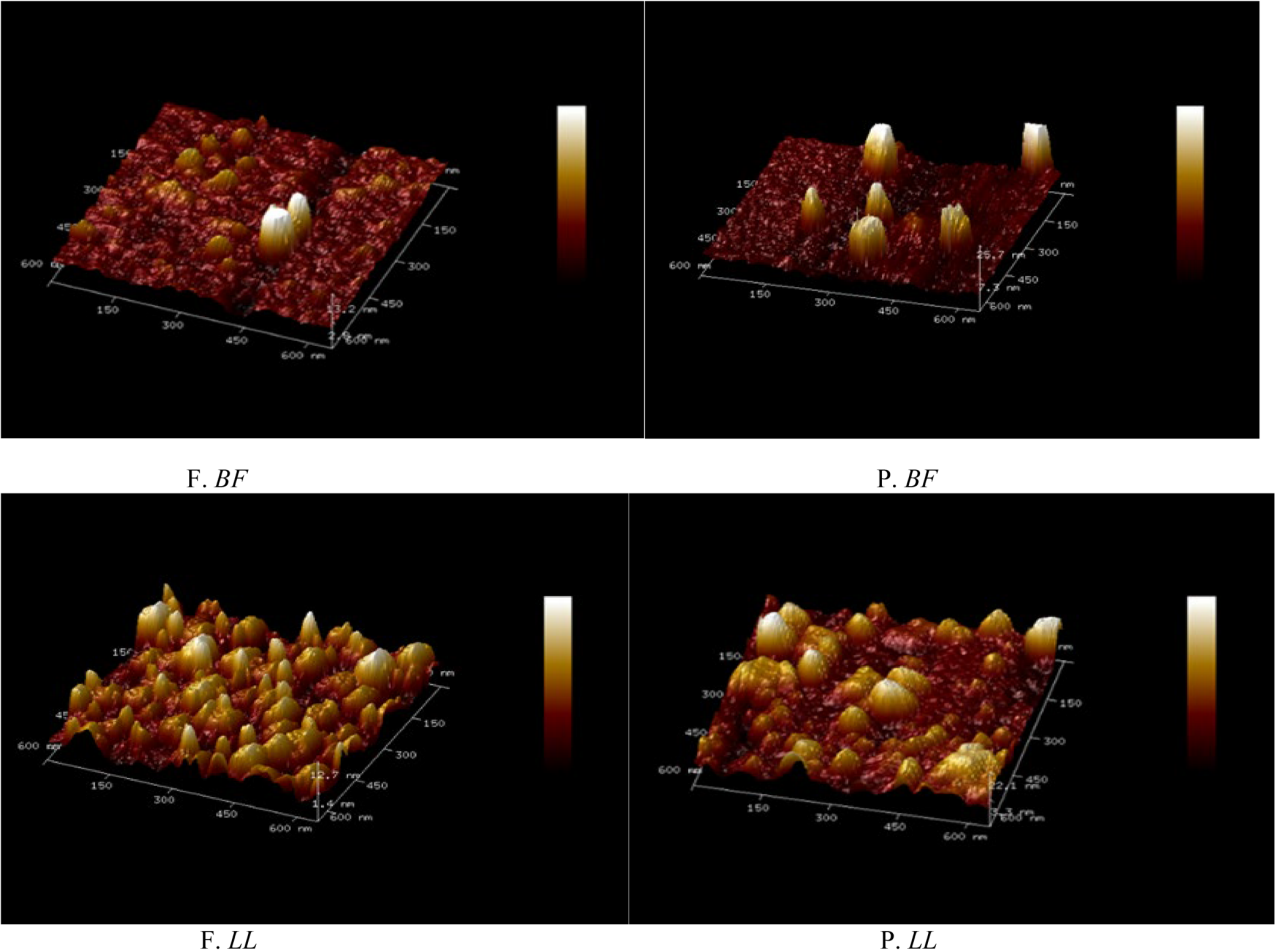
Tapping‐mode AFM images of *BF* and *LL* myofibrillar proteins under fresh and preserved conditions. 3D reconstructions (650 × 650 nm) show surface morphology and nanoscale topography.

The tapping‐mode AFM images and corresponding three‐dimensional reconstructions (650 × 650 nm) presented in Figure 3 further confirmed these observations. Fresh samples (F. *BF* and F. *LL*) displayed rounded morphologies with relatively uniform height distribution and clearly defined edges (Figure 3). In contrast, preserved samples showed heterogeneous height distribution, fragmented domains, and irregular protrusions, particularly in P.*LL* (Figure), indicating nanoscale structural disruption.

**FIGURE 4:**
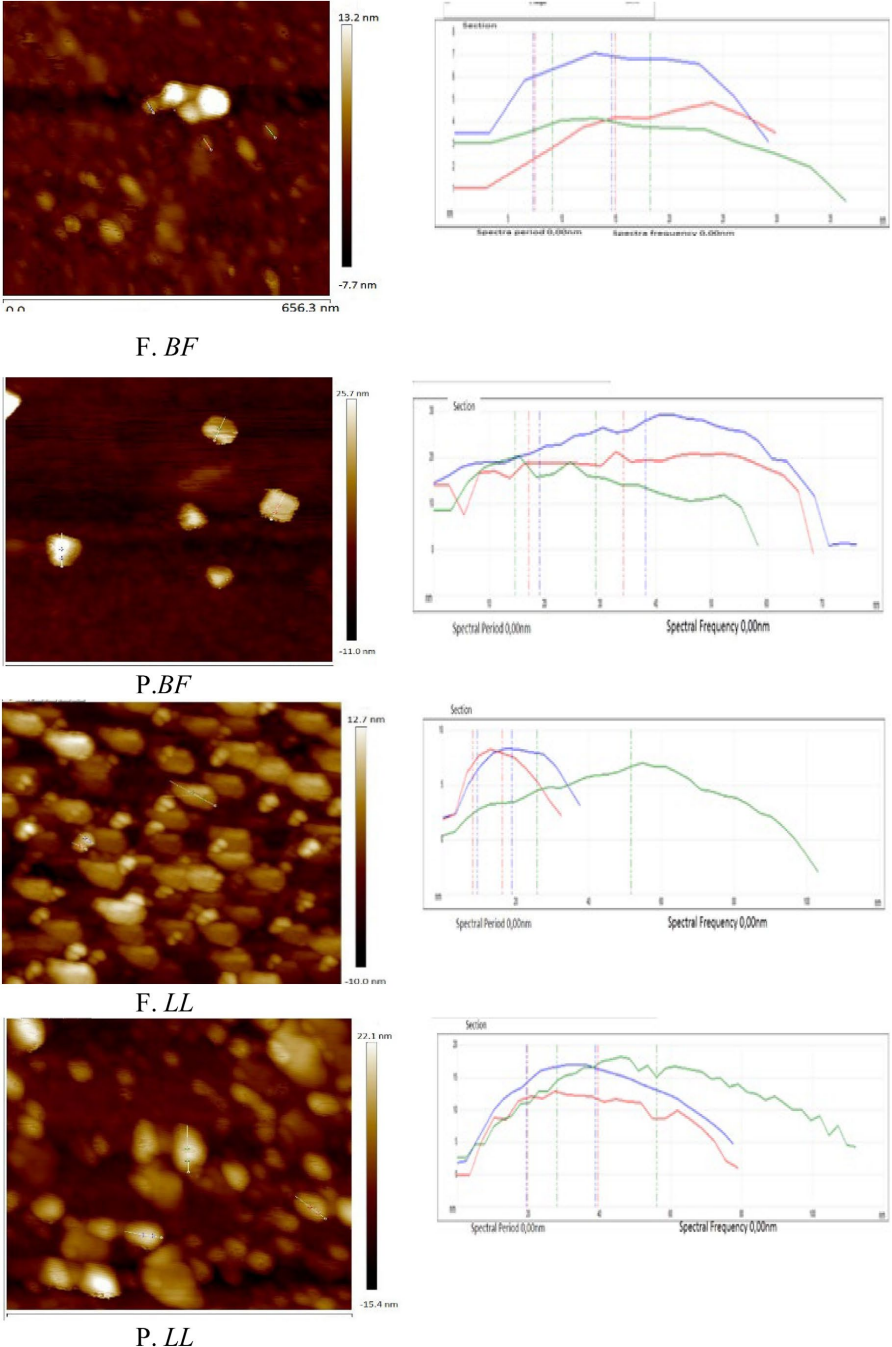
High‐resolution AFM images of myofibrillar proteins from *Biceps femoris* (BF) and *Longissimus lumborum* (LL) muscles under fresh (F) and preserved (P, 1 year at −20°C) conditions. The blue, green, and red lines inside the images indicate the cross‐sectional profiles (650 × 650 nm) used for width measurements (Table 2). A higher magnification view allows these lines to be clearly observed.

To quantitatively support these surface observations, high‐resolution AFM images were analyzed (Figure 4). The blue, green, and red lines drawn across the protein particles represent the cross‐sectional profiles used for width measurements (Figure 4).

Fresh *BF* samples exhibited relatively narrow cross‐sectional widths (28.87–35.52 nm), whereas preserved *BF* samples showed markedly increased widths (58.48–77.14 nm) (Figure 4; Table 2). Similarly, fresh *LL* samples displayed widths ranging from 33.74 to 101.03 nm, which increased to 78.66–112.41 nm after frozen storage (Figure 4; Table 2). The enlargement of cross‐sectional dimensions observed in preserved samples was consistent with the surface irregularities and aggregation patterns detected in Figures 2 and 3. Notably, preserved *LL* exhibited greater structural expansion compared to preserved *BF* (Figure 4; Table 2).

**TABLE 2:** Cross‐sectional width measurements (nm) of myofibrillar proteins extracted from AFM line profiles (blue, green, and red lines) of *Biceps femoris* (BF) and *Longissimus lumborum* (LL) muscles under fresh and frozen (−20°C, 1 year) conditions (Figure 4), and after thermal treatment (Figure 6). Values represent representative nanoscale measurements obtained from AFM cross‐sectional analysis.

Protein		Spectral frequency	Width (nm)	Width (after heating, nm)
*BF*	Fresh	Blue Line	28.87	55.06
		Green Line	35.52	60.27
		Red Line	30.27	61.18
	Preserved	Blue Line	77.14	182.26
		Green Line	58.48	121.35
		Red Line	66.46	131.56
*LL*	Fresh	Blue Line	37.84	111.85
		Green Line	101.03	221.37
		Red Line	33.74	149.94
	Preserved	Blue Line	78.66	232.23
		Green Line	112.41	270.55
		Red Line	80.81	217.59

#### 3.3.2 Nanoscale Structural Characterization of Thermally Treated Myofibrillar Protein Gels

**FIGURE 5:**
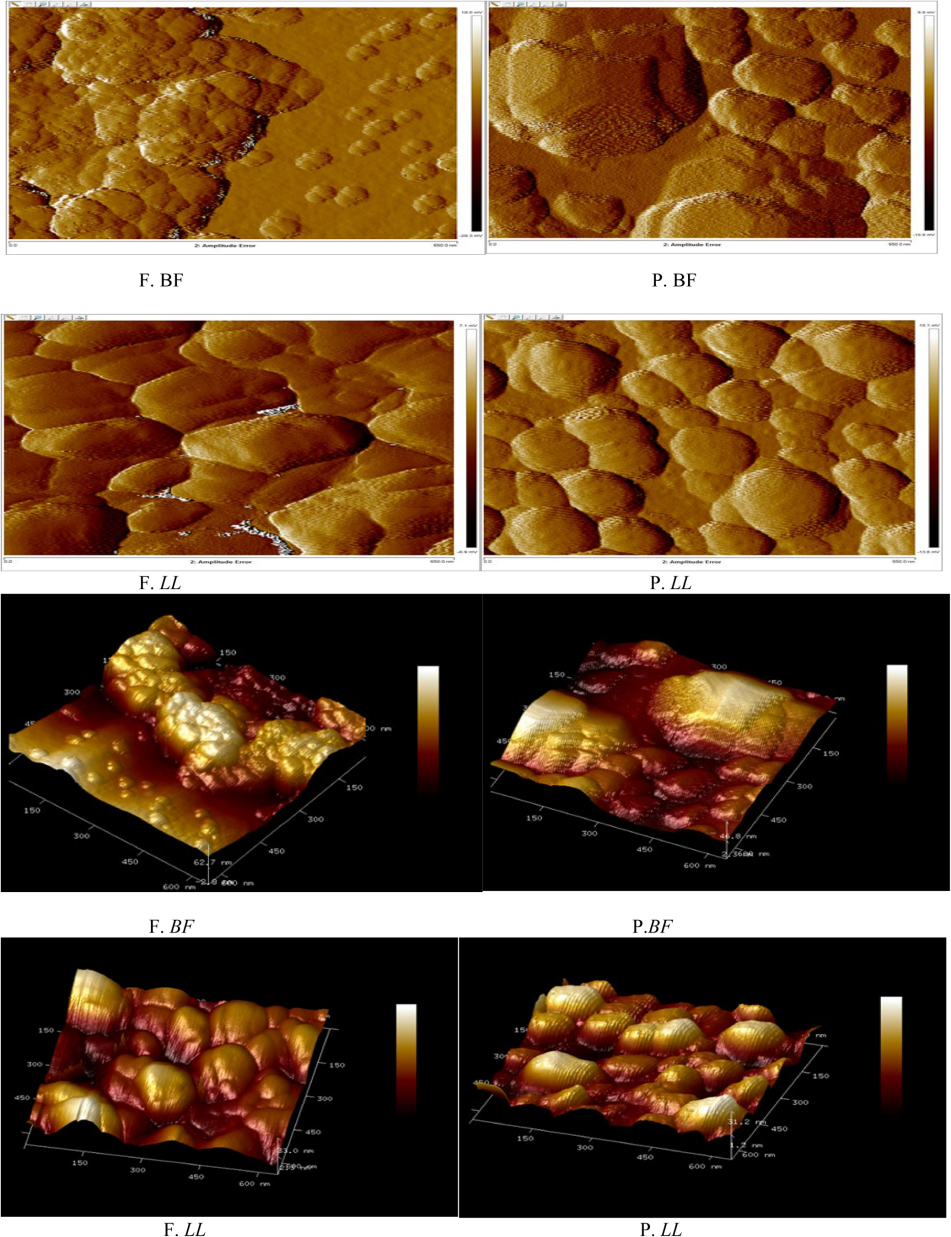
Tapping mode AFM image of individual proteins from different muscle types after thermal treatment (70°C) with corresponding 3D reconstructions (650 × 650 nm).

**FIGURE 6:**
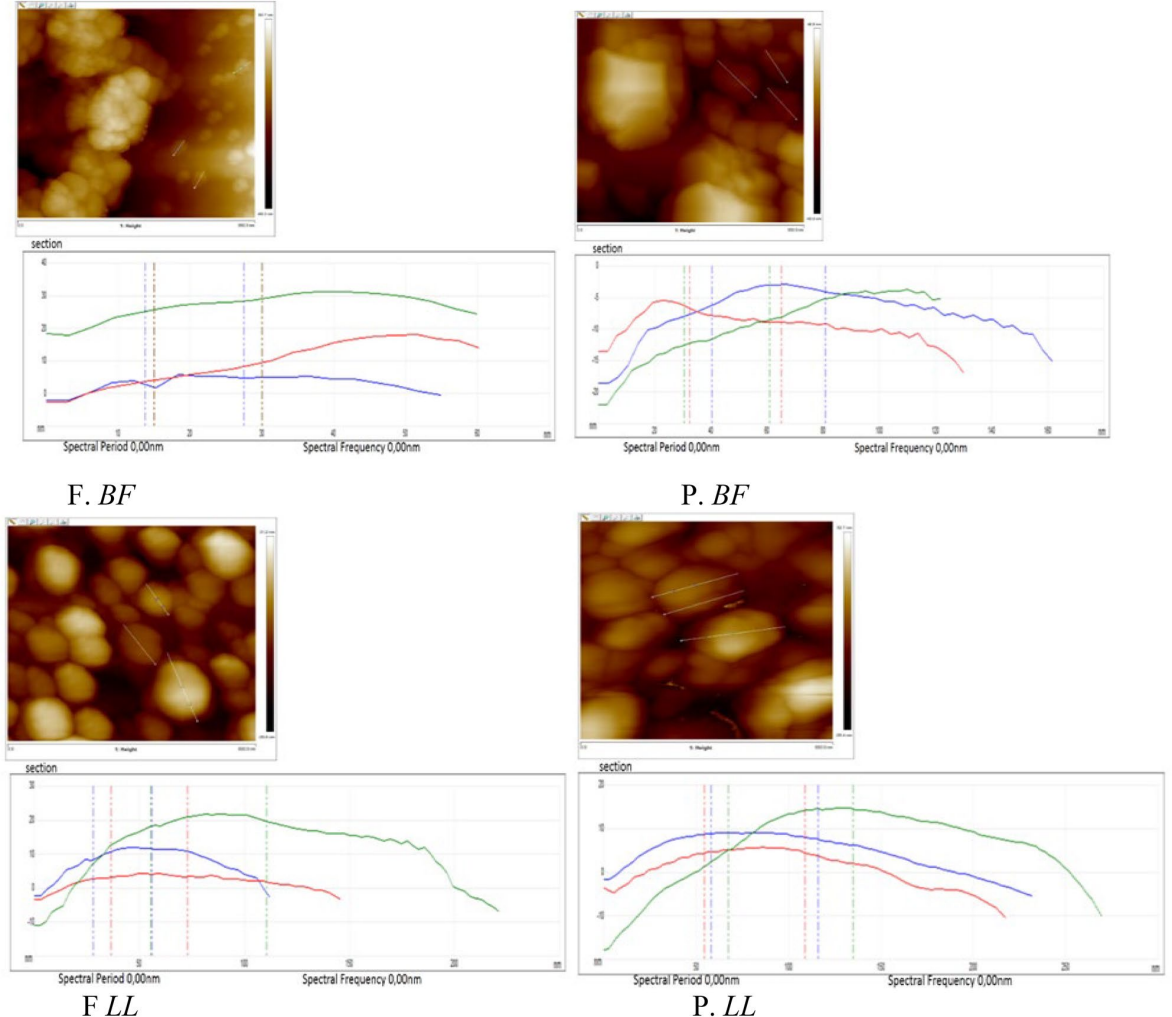
High‐resolution AFM images of myofibrillar proteins from *Biceps femoris* (BF) and *Longissimus lumborum* (LL) muscles after thermal treatment (70°C) under fresh (F) and preserved (P, 1 year at −20°C) conditions. The blue, green, and red lines within the images represent the cross‐sectional profiles (650 × 650 nm) used for width measurements (Table 2). These lines become clearly visible at higher magnification.

AFM images of myofibrillar protein (MP) gels after thermal treatment at 70°C are presented in Figures 5 and 6. Distinct structural differences were observed between fresh and preserved samples.

In fresh *BF* and *LL* gels, AFM images revealed relatively continuous and compact gel networks with interconnected protein assemblies (Figure 5, F. *BF* and F. *LL*). The surface morphology appeared moderately uniform, with organized aggregation patterns and limited surface irregularities. The corresponding three‐dimensional reconstructions showed cohesive network structures with controlled height distribution (Figure 5). Following frozen storage and subsequent heating, preserved *BF* and *LL* gels exhibited marked structural modifications. AFM images revealed enhanced aggregation, irregular clustering, lateral expansion of protein assemblies, and visible surface heterogeneity (Figure 5, P. *BF* and P. *LL*). These structural disruptions were more pronounced in preserved LL gels, where large protrusions and heterogeneous surface domains were observed. The high‐resolution AFM images further detailed these nanoscale alterations (Figure 6). Fresh gels displayed relatively compact aggregates with distinguishable boundaries (Figure 6, F. *BF* and F. *LL*). In contrast, preserved gels showed enlarged aggregates, fragmented regions, and irregular surface topology (Figure 6, P. *BF* and P. *LL*).

To quantitatively evaluate these changes, cross‐sectional profiles were extracted from the high‐resolution AFM images (Figure 6), and aggregate width values were calculated (Table 2).

In *BF* gels, cross‐sectional widths increased from 55.06–61.18 nm in fresh samples to 121.35–182.26 nm after frozen storage and heating (Figure 6; Table 2). Similarly, in *LL* gels, aggregate widths increased from 111.85–221.37 nm in fresh samples to 217.59–270.55 nm following preservation and thermal treatment (Figure 6; Table 2). Among all treatments, preserved *LL* gels exhibited the largest aggregate dimensions and the most heterogeneous structural organization (Figure 6; Table 2).

## 4 Discussion

### 4.1 Biochemical Changes in Myofibrillar Proteins

This study demonstrates the significant increase in protein solubility observed after long‐term frozen storage indicates that freezing induced marked structural alterations in myofibrillar protein (MP) particles. Similar modifications in protein extractability following freezing and thawing have been reported by Chan et al. (2011), Qi et al. (2012), and Tuell et al. (2020). (Chan et al. 2011; Qi et al. 2012; Tuell et al. 2020; Wu et al. 2024; Zhu et al. 2025). While many studies describe reductions in solubility due to aggregation phenomena, Chan et al. (2011) reported an increase in solubility in turkey breast meat after freezing and thawing, consistent with the present findings. Freeze‐induced denaturation of muscle proteins (Benjakul and Bauer 2000; Benjakul et al. 2003; Zhu et al. 2025) may disrupt protein–protein interactions and modify conformational stability, thereby altering extractability. Drip loss during thawing can also release sarcoplasmic proteins into the exudate, contributing to increased measurable solubility. Moreover, structural rearrangements during freezing may reduce surface hydrophobic exposure, favoring greater interaction with surrounding water molecules, as suggested by Fennema (1996) (Fennema 1996). Freezing‐induced modifications in enzymatic systems, such as alterations in Ca^2+^‐ATPase activity (Jiang et al. 2019; Li et al. 2018), further support the occurrence of structural and membrane‐related destabilization during frozen storage.

Concurrently, the marked increase in surface hydrophobicity (SurH) observed after preservation reflects protein unfolding and exposure of hydrophobic domains. Similar increases in SurH following freezing and thawing have been documented in muscle systems (Qian et al. 2019; Xia et al. 2009, 2010; Zhang and Ertbjerg 2019). According to Lin and Park (1998), myosin filaments possess a hydrophilic outer surface and a hydrophobic inner core; disruption of this structural organization during freezing may expose buried hydrophobic groups, thereby increasing measured SurH (Lin and Park 1998). In addition, pH‐dependent conformational shifts can influence hydrophobic exposure, as demonstrated by Chan et al. (2011) and Omana et al. (2010), who reported higher SurH in high‐pH meat due to greater protein unfolding (Chan et al. 2011; Omana et al. 2010). The present increase in SurH after frozen storage is therefore consistent with mild to moderate denaturation processes, as also described by Farouk et al. (2004), Lee et al. (2024), and Zhang et al. (2022) (Farouk et al. 2004; Lee et al. 2024; Zhang et al. 2022).

At the same time, the significant reduction in reactive sulfhydryl (R–SH) content indicates oxidative and conformational modifications of MPs during long‐term freezing. Sulfhydryl groups are highly reactive and particularly susceptible to oxidative reactions, leading to disulfide bond formation either within or between polypeptide chains (Xia et al. 2009). Similar decreases in R–SH and total sulfhydryl groups during frozen storage have been reported by Chan et al. (2011), Soyer et al. (2010), and Liu et al. (2022) (Chan et al. 2011; Liu et al. 2022; Soyer et al. 2010). Oxidation of cysteine residues reduces thiol accessibility and promotes aggregation, thereby altering protein functionality. Moreover, pH‐induced structural compaction can further limit thiol exposure (Chan et al. 2011), contributing to the observed reduction in R–SH.

### 4.2 Muscle‐Specific Myofibrillar Protein Alterations Revealed by SDS–PAGE

The SDS–PAGE profiles revealed clear muscle‐specific differences in the response to long‐term frozen storage in *Biceps femoris* (*BF*) and *Longissimus lumborum* (*LL*) muscles of *Camelus dromedarius* . While *BF* exhibited relatively minor reductions in major myofibrillar proteins, *LL* showed pronounced degradation of MHC, α‐actinin, actin, troponin T (TT), and myosin light chains, along with the appearance of additional bands between 75 and 85 kDa.

The reduction in MHC intensity and the appearance of lower molecular weight fragments in *LL* indicate partial degradation of high‐molecular‐weight myofibrillar proteins. Similar electrophoretic changes have been reported in frozen red meats and were attributed to storage‐induced denaturation and fragmentation (Park et al. 2007; Zhang et al. 2021). Previous studies on camel muscle also identified comparable major myofibrillar bands (Maqsood et al. 2015, 2016, 2018; Wu et al. 2024), supporting the assignment of the degraded proteins observed in the present study.

The marked disappearance of TT, particularly in *LL*, further confirms its high susceptibility to postmortem proteolysis. Although TT degradation has been proposed as an indicator of proteolytic activity, no consistent positive relationship has been established between TT loss and meat tenderness (Reza Gheisari et al. 2009). Therefore, the pronounced TT reduction observed here should be interpreted as evidence of structural protein modification rather than functional improvement.

The more extensive degradation observed in *LL* compared to *BF* likely reflects intrinsic muscle characteristics. *LL* contains a higher proportion of oxidative type I fibers, whereas *BF* is richer in glycolytic type II fibers (Brandstetter et al. 1998; Geay et al. 2001). Oxidative muscles generally exhibit higher myoglobin content and metabolic activity, increasing their susceptibility to oxidative reactions during storage (Faustman 1993; Faustman et al. 2010). Oxidative stress has been shown to promote myosin modification and fragmentation (Andersen et al. 2007; Park et al. 2007; Wu et al. 2024), which aligns with the stronger MHC degradation observed in *LL* in this study.

Additionally, freezing‐induced structural damage may enhance the release and activity of endogenous proteases such as calpains and cathepsins (Coria‐Hernández and Meléndez‐Pérez 2024; Geesink et al. 2006; Koohmaraie and Geesink 2006). Differences in muscle structure and water‐holding properties between *LL* and *BF* could influence the extent of ice crystal formation and mechanical disruption (Leygonie et al. 2012a, 2012b), thereby contributing to the distinct electrophoretic patterns observed. These findings highlight that protein stability during prolonged frozen storage is not uniform across muscles and is strongly influenced by their intrinsic biochemical and structural characteristics, as evidenced by the markedly greater degradation of MHC, TT, and MLC observed in *LL* compared to *BF*.

### 4.3 AFM Structural Modifications

The AFM observations indicate that long‐term frozen storage induces significant nanoscale structural alterations in camel myofibrillar proteins. The appearance of concave depressions, increased surface roughness, disrupted fibrillar alignment, and enlarged cross‐sectional dimensions suggest partial unfolding and aggregation of protein structures. Similar patterns of surface irregularity and structural fragmentation have been reported in frozen lamb (Muela et al. 2015, 2016; Muela et al. 2010, 2012) and beef (Wang et al. 2024), where freezing disrupted protein–protein and protein–water interactions.

The structural enlargement quantified by cross‐sectional analysis is consistent with protein denaturation and aggregation phenomena previously described in frozen meat systems (Moczkowska et al. 2017; Nawaz et al. 2022). Morphological transitions observed at the nanoscale may reflect accelerated unfolding and formation of extended aggregates under environmental stress, involving alterations in hydrogen bonding and increased intermolecular interactions (Li et al. 2024, 2023).

The more extensive disorganization detected in *LL* muscle compared to *BF* aligns with reported muscle‐specific variations in connective tissue density and sarcomere stability (Adeyemi et al. 2016; Sikorski and Kołakowska 1994; Xiong 1994). Such differences may explain the greater susceptibility of *LL* proteins to freeze‐induced structural damage.

Thermal treatment further intensified molecular reorganization, promoting aggregation and modification of the gel network (Coria‐Hernández and Meléndez‐Pérez 2024; Nanje et al. 2024). Similar structural transitions during heating have been documented in food protein and hydrocolloid systems (Hedayati et al. 2022; Mills et al. 2001; Zhang et al. 2024; Zielbauer et al. 2016). The formation of irregular aggregates during heating has been attributed to exposure of reactive groups and enhanced intermolecular interactions (Lee et al. 2024; Mackie 1993).

The distorted surface topographies observed in preserved samples, particularly in *LL*, resemble nanoscale disruptions previously described in frozen fish muscles subjected to subzero storage (Pornrat et al. 2007). Comparable nano‐architectural alterations have also been reported using high‐resolution AFM in muscle protein systems (Ding et al. 2019; Fuentes‐Perez et al. 2013; Jiang et al. 2025; Shi et al. 2019a, 2019b).

Hu et al. (2021) demonstrated that phosphate‐treated MPs exhibited improved structural organization and gel formation (Hu et al. 2021). In contrast, the absence of stabilizing additives in the present study may have contributed to the more heterogeneous and enlarged aggregate structures observed after frozen storage and subsequent heating. The cumulative structural modifications observed here are consistent with progressive destabilization of myofibrillar protein architecture during frozen storage and thermal processing.

### 4.4 General Discussion: Integrated Multi‐Scale Mechanistic Interpretation

This study provides an integrated multi‐scale interpretation of the structural evolution of camel myofibrillar proteins during long‐term frozen storage and subsequent heating. By combining biochemical analyses (solubility, surface hydrophobicity, reactive sulfhydryl content), SDS‐PAGE profiling, and AFM nanoimaging, the results describe a hierarchical progression linking molecular oxidation and unfolding to supramolecular aggregation and gel network disruption (Huff‐Lonergan and Lonergan 2005; Xiong 1994).

Frozen storage induced oxidative modifications, evidenced by increased surface hydrophobicity and decreased reactive sulfhydryl content, indicating exposure of buried residues and thiol oxidation with potential disulfide bond formation (Xia et al. 2009). SDS‐PAGE further confirmed molecular destabilization through partial degradation and reduced band intensity of major myofibrillar proteins. Although solubility increased during storage, this likely reflects enhanced extractability of partially unfolded proteins rather than improved structural stability, highlighting differences in sensitivity among analytical approaches.

AFM observations provided nanoscale confirmation of these molecular events. Preserved samples exhibited enlarged aggregates, increased surface roughness, disrupted fibrillar alignment, and expanded cross‐sectional dimensions, directly visualizing aggregation phenomena inferred from biochemical and electrophoretic data (Dong et al. 2020; Jiang et al. 2025). These structural changes are consistent with oxidative unfolding promoting hydrophobic interactions and disulfide cross‐linking.

Thermal treatment at 70°C further intensified aggregation, producing coarser and more heterogeneous gel network structures (Coria‐Hernández and Meléndez‐Pérez 2024), particularly in previously frozen samples (Mackie 1993; Nanje et al. 2024). The combined effects of freezing and heating suggest progression toward irreversible aggregation pathways and reduced structural compactness.

Muscle‐dependent differences were evident, with *LL* samples showing greater sulfhydryl depletion, stronger aggregation behavior, and more pronounced nanoscale disruption. These findings provide direct experimental support for higher oxidative susceptibility in *LL* and strengthen the mechanistic linkage between intrinsic muscle characteristics and structural destabilization.

Overall, the strong agreement between biochemical indicators, electrophoretic patterns, and AFM nano‐architecture supports a coherent mechanistic sequence in which frozen storage initiates oxidative unfolding and molecular destabilization, leading to aggregation and supramolecular reorganization that are further amplified by heating. This multi‐scale integration reinforces the robustness of the interpretation and its relevance for understanding muscle protein functionality during frozen storage and thermal processing.

### 4.5 Limitations

The study presented here focused on a single prolonged frozen storage condition (1 year at −20°C), which limits the scope of interpretation. As only one storage duration and temperature were evaluated, the results cannot establish a comprehensive time‐dependent or temperature‐dependent relationship. Commercial meat supply chains frequently involve shorter storage periods and variable temperature conditions. Therefore, the findings should be interpreted as reflecting structural and molecular responses to extended frozen storage rather than representing the full diversity of industrial preservation practices.

The study did not include direct measurements of classical techno‐functional parameters such as water‐holding capacity or instrumental texture. Consequently, cause‐and‐effect relationships between molecular alterations and macroscopic quality attributes cannot be definitively established. Instead, the investigation centered on biochemical and nano‐structural indicators—including protein solubility, surface hydrophobicity, reactive sulfhydryl content, electrophoretic patterns, and AFM morphology—which are widely recognized as sensitive markers of protein denaturation and aggregation. Thus, the primary objective was to characterize structural evolution at multiple analytical scales rather than to quantify final product performance.

Only two camel muscles, Biceps femoris (BF) and Longissimus lumborum (LL), were examined. Although these muscles differ in metabolic and structural characteristics and are commercially relevant cuts, they do not represent the full range of fiber composition present in the camel carcass. The study did not aim to provide a comprehensive characterization of all camel muscles, but rather to compare contrasting muscle types in order to highlight muscle‐dependent responses to long‐term freezing at molecular and nano‐structural levels.

### 4.6 Future Directions

The findings of the present study open several avenues for further research aimed at strengthening both mechanistic understanding and industrial applicability. Because the experimental design focused exclusively on a single prolonged frozen storage period (1 year at −20°C), the temporal evolution of protein destabilization could not be fully characterized. Future investigations incorporating shorter and intermediate storage durations (e.g., 1, 3, and 6 months) would allow a clearer distinction between immediate freezing‐induced alterations and progressive storage‐related oxidative or aggregation phenomena. Such an approach would clarify whether the structural modifications observed in this study develop gradually, reach a plateau, or follow a nonlinear progression under conditions that more closely reflect commercial practice.

Future work should also integrate classical techno‐functional measurements, including water‐holding capacity, cooking loss, gel strength, and instrumental texture. Combining these macroscopic parameters with molecular and nano‐structural indicators would enable stronger structure–function correlations and improve the predictive value of biochemical markers for meat quality assessment.

Expanding the analysis to additional muscles with varying anatomical and metabolic characteristics would enhance generalizability and provide a broader understanding of muscle‐specific responses to frozen storage.

## 5 Conclusion

In this study, integrated biochemical, electrophoretic, and nanoscale analyses were performed to elucidate the structural evolution of camel myofibrillar proteins during long‐term frozen storage (−20°C for 1 year) and subsequent thermal treatment. The results demonstrated that frozen storage significantly modified protein conformation and stability, as evidenced by increased surface hydrophobicity, reduced reactive sulfhydryl content, and altered electrophoretic profiles of major myofibrillar proteins. Partial degradation of MHC, TT, and MLC was particularly pronounced in *Longissimus lumborum* compared with *Biceps femoris*. AFM observations further confirmed substantial nanoscale reorganization, including increased surface roughness, disrupted fibrillar alignment, and enlarged aggregate structures, especially in preserved and heated samples. Thermal processing intensified aggregation phenomena, indicating that freezing‐induced molecular destabilization predisposes proteins to further structural rearrangement during heating.

Overall, long‐term frozen storage followed by heating induces progressive oxidative and conformational changes that compromise the structural integrity of camel myofibrillar systems, with muscle‐specific susceptibility clearly demonstrated. These findings provide a structural basis for understanding quality changes in frozen camel meat and offer valuable guidance for optimizing storage duration and processing conditions in industrial applications.

## Author Contributions

**Ramdani Nacira:** data curation, investigation, methodology, funding acquisition. **Bendjaballah Sandra:** data curation, software, formal analysis. **Cherb Nora:** data curation, supervision, formal analysis, methodology. **Rahmani Abderrahmen:** data curation, investigation, methodology, resources. **Ahmed‐Laloui Hamza:** conceptualization, project administration, resources, validation, visualization, writing – original draft. **Seid Mahdi Jafari:** formal analysis, methodology, validation, visualization, writing – review and editing. **Sajid Maqsood:** data curation, visualization, validation, supervision.

## Funding

The authors have nothing to report.

## Conflicts of Interest

The authors declare no conflicts of interest.

## Data Availability

The data that support the findings of this study are available from the corresponding author upon reasonable request.
