# Correction to “Impact of Freezing on the Nanoarchitecture and Techno‐Functional Properties of Camel Myofibrillar Proteins: Insights From Atomic Force Microscopy”

**DOI:** 10.1002/fsn3.72400

**Published:** 2026-09-24

Hamza, A.‐L. , R. Abderrahmen , C. Nora , et al. 2026. “Impact of Freezing on the Nanoarchitecture and Techno‐Functional Properties of Camel Myofibrillar Proteins: Insights From Atomic Force Microscopy.” Food Science & Nutrition 14, no. 5: e71668. 10.1002/fsn3.71668.PMC1312156742058721

The name of the sixth author was incorrect and has been updated from Maqsood Sajid to Sajid Maqsood.

The online version of this article is corrected accordingly.

We apologize for this error.

